# Adequacy of prenatal care and associated factors in the State of Rio Grande do Sul, Brazil

**DOI:** 10.11606/s1518-8787.2023057005146

**Published:** 2023-09-14

**Authors:** Aline De Gregori Adami, Leonardo Rapone da Motta, Rosa Dea Sperhacke, Sérgio Kakuta Kato, Gerson Fernando Mendes Pereira, Rosa Maria Rahmi

**Affiliations:** I Universidade de Caxias do Sul Programa de Pós-graduação em Ciências da Saúde Caxias do Sul RS Brazil Universidade de Caxias do Sul. Programa de Pós-graduação em Ciências da Saúde. Caxias do Sul, RS, Brazil.; II Universidade de Caxias do Sul Instituto de Pesquisas em Saúde Área do Conhecimento de Ciências da Vida Caxias do Sul RS Brazil Universidade de Caxias do Sul. Instituto de Pesquisas em Saúde. Área do Conhecimento de Ciências da Vida. Caxias do Sul, RS, Brazil.; III Universidade Federal de Ciências da Saúde de Porto Alegre Departamento de Saúde Coletiva Porto Alegre RS Brazil Universidade Federal de Ciências da Saúde de Porto Alegre. Departamento de Saúde Coletiva. Porto Alegre, RS, Brazil.; IV Ministério da Saúde Secretaria de Vigilância em Saúde Departamento de HIV/aids, Tuberculose, Hepatites Virais e Infecções Sexualmente Transmissíveis Brasília DF Brazil Ministério da Saúde. Secretaria de Vigilância em Saúde. Departamento de HIV/aids, Tuberculose, Hepatites Virais e Infecções Sexualmente Transmissíveis. Brasília, DF, Brazil.

**Keywords:** Prenatal Care, Health Services Coverage, Maternal Health Services, Health Care Quality, Access, and Evaluation, Delivery, Obstetric

## Abstract

**OBJECTIVE:**

This study aims to assess the degree of adequacy of prenatal care (PNC) in the state of Rio Grande do Sul (RS) and in its 7 macro-regions considering the time of PNC initiation and the number of appointments attended. It also aims to verify the mode of delivery prevalence and the factors associated with PNC adequacy by mode of delivery.

**METHODS:**

Sub analysis from a cross-sectional study conducted among 13,432 childbearing women aged 15–49 years assisted in 66 maternity hospitals of the Unified Health System (SUS) and private associated facilities from September 2017 to October 2019. A standardized form was used to collect sociodemographic data, and information about PNC and delivery from the childbearing women’s prenatal cards, hospital records, and medical reports.

**RESULTS:**

The PNC coverage was (98.4%), but only 57.5% of the participants had an adequate PNC defined as the one initiated until the 12th gestational week, with attendance of at least 6 appointments. The cesarean rate was 57.2%. Among women who performed vaginal delivery, multivariate analysis showed that for each 1-year increase in the age of the parturient, the chance of having an adequate PNC increased by 5%. White parturients with higher education and fewer deliveries residing in the macro-region of Valleys were more likely to have an adequate PNC when compared with non-white parturients, who were illiterate and/or had incomplete elementary school, with 3 or more deliveries and who resided in other macro-regions. During pregnancy, 96.0% of the women performed at least one anti-HIV test, 55.8% a rapid test for syphilis, and 75.0% a Venereal Disease Research Laboratory test (VDRL).

**CONCLUSIONS:**

Despite the almost universal PNC coverage in RS, the PNC offered by the SUS was adequate for just half of the population, therefore public health policies targeted at women receiving care in this setting shall be revisited.

## INTRODUCTION

The World Health Organization defines prenatal care (PNC) as a set of health practices provided by skilled health care professionals to pregnant women and adolescent girls to ensure the best health conditions for the development of the pregnancy, allowing the delivery of a healthy newborn, without impacting on maternal health^1^. It includes addressing psychosocial aspects, identification of risk, prevention and management of pregnancy-related or concurrent diseases, and promotion of health education and other preventive activities^1,2^.

Maternal and infant mortalities are associated with the PNC inadequacy^3^. Therefore, a PNC with early onset and intervention for risk situations, as well as an agile hospital referral system, and the qualification of childbirth care, are the major health determinant indicators related to the mother and the newborn^3,4^.

In 2011, the Brazilian Ministry of Health (BMoH), published an ordinance establishing the *Rede Cegonha* (Stork Network). This network aims to ensure women the right of receiving humanized reproductive care during pregnancy planning, pregnancy, childbirth, and the puerperium period, as well as promoting a safe birth and the growth and development of healthy neonates^6^. In this ordinance, an adequate PNC is defined as initiated until the 12th gestational week, with attendance of at least 6 appointments distributed throughout pregnancy’s trimesters^6^.

From 2017 to 2019, approximately 416,211 live births occurred in the state of Rio Grande do Sul (RS), Brazil^7^. In 2021, the RS presented the highest HIV detection rate in pregnant women (8.4 cases/1,000 live births) nationwide^8^, the second highest rate of syphilis (38.1/1,000 live births), and the fifth highest incidence rate of congenital syphilis, with 15.8 cases/1,000 live births, higher than the national rate^9^. These data suggest an inadequacy in PNC in RS.

The PNC is the ideal timeframe for health interventions to be carried out. The sooner the monitoring of pregnant women begins, the greater the chances they will receive adequate counselling, perform more consultations and complementary exams, and, consequently, have the possibility of properly identifying and treating the health problems. By qualifying the PNC, observing the reflexes of this intervention in reducing the detection rates of HIV/AIDS, syphilis, and congenital syphilis in pregnant women will be possible, for instance.

Therefore, data regarding the adequacy of PNC and the profile of women receiving care in maternity hospitals of the Unified Health System (SUS) and private associated facilities in RS is relevant to assess and improve public health policies targeted at women receiving care in this setting.

This article aims to present the degree of adequacy to PNC in RS and in its 7 macro-regions (namely, North, South, Midwest, Sierra, Valleys, Missionary, and Metropolitan), according to the BMoH guidelines^6,10^ which consider the time of PNC initiation and the number of appointments attended. It also aims to verify the mode of delivery prevalence and the factors associated to the PNC adequacy by mode of delivery.

## METHODS

### Ethics

This study was approved by the local Institutional Committee of Ethics in Research at Universidade de Caxias do Sul (Caxias do Sul, RS, Brazil), register number 868.345 on November 17, 2014. All participants signed a written informed consent form.

### Study Design, Subject Selection, and Sampling

This article is a sub analysis of survey data obtained from a macro-project entitled: “*Projeto de verificação da prevalência do HIV e sífilis em parturientes no Rio Grande do Sul a partir de dados secundários (cartão pré-natal e prontuário)*” (Project to verify the prevalence of HIV and syphilis in pregnant women in Rio Grande do Sul based on secondary data [prenatal cards, medical records, and reports]) – *Projeto Parturientes RS*. The *Projeto Parturientes RS* was a cross-sectional study conducted with childbearing women aged 15 to 49 years who gave birth in public maternity hospitals or private associated facilities from September 2017 to October 2019.

The sample size was calculated to estimate the HIV prevalence in childbearing women residing in RS, Brazil. Based on 2010/2011 estimates of HIV infection in pregnant women, 0.79%^11^, a sample size of 14,300 pregnant women, distributed throughout the seven macro-regions of the State, was calculated to estimate HIV infection with a 95% confidence interval, bilateral error of 0.15%, and a design effect of 1.1.

A stratified two-stage cluster sampling design was used. In the first stage, 66 public maternity hospitals or private associated facilities representing all seven macro-regions of the state (namely, North, South, Midwest, Sierra, Valleys, Missionary, and Metropolitan) were selected to recruit childbearing women for this study (Figure). To be eligible for selection, hospitals should have performed more than 350 births in 2012. The population size of the municipality in which the maternity hospital was located (less than 80,000 inhabitants, 80,000 to 199,999 inhabitants, and equal to or greater than 200,000 inhabitants), and their geographical macro-regions was used to stratify the health establishments. In each stratum, health establishments were selected with probability proportional to size, as established by the number of admissions for delivery in 2012. Within each maternity hospital, 200–500 childbearing women were selected upon admission.

**Figure:**
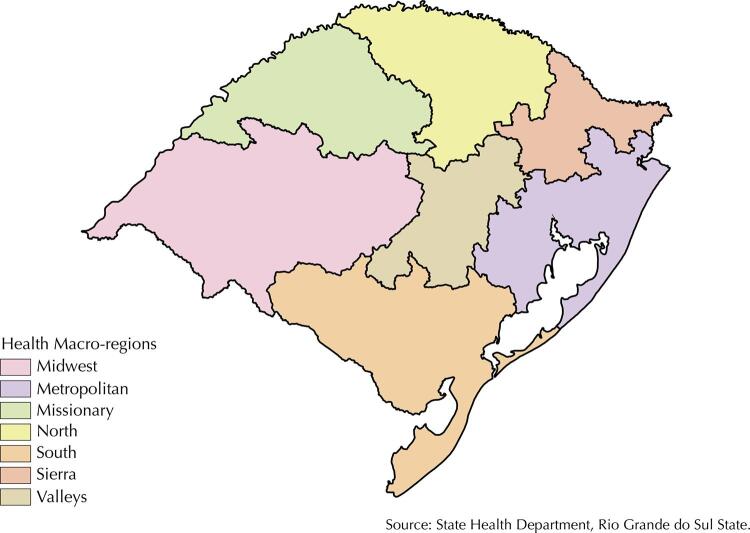
Health Macro-regions Map: State of Rio Grande do Sul, Brazil.

The exclusion criteria for this study were as follows: pregnant women hospitalized for post-abortion curettage; pregnant women outside the age range of 15–49 years; pregnant women who did not reside in the RS; and those who refused to provide informed consent.

### Data Collection

A standardized form was used to collect sociodemographic data (date of birth, level of education, race/ethnicity, municipality of residence) and information about PNC and delivery, including number of PNC visits, date of first PNC visit, gestational age at the first PNC visit, date of last menstrual period, municipality and health care unit where PNC was carried on, results and dates of syphilis and HIV tests, number of previous pregnancies, date and mode of delivery, and gestational age at birth. Information was gathered from the childbearing women’s prenatal cards, hospital records, and medical reports, when available.

All questionnaires were processed at the Instituto de Pesquisas em Saúde (Universidade de Caxias do Sul, Caxias do Sul, RS, Brazil) using OpenText^®^ TeleForm 16.2 (Waterloo, Ontario, Canada).

### Statistical Analysis

All analyses were performed using the IBM SPSS Statistics for Windows software, version 22.0 (IBM Corp, Armonk, USA), with a significance level of 5%. The analyses incorporated data weighting, clustering, and stratification. Since the dataset was obtained by using a complex sampling procedure that combined stratification and clustering, the design of the survey was incorporated into the statistical analysis of the data. Additionally, a calibration procedure was applied for the samples according to the census distribution by population size of the city of residence (less than 80,000 inhabitants, 80,000 to 199,999 inhabitants, and equal to or greater than 200,000 inhabitants).

Qualitative variables were presented as absolute and relative frequencies, and quantitative variables were presented as means and standard deviations (SD). The nonparametric Mann-Whitney U-Test was used to compare levels of education with PNC adequacy. The PNC adequacy and mode of delivery prevalence were expressed with a binomial 95% confidence interval (95%CI). Multivariate logistic regression analysis was used to investigate the factors mostly associated with the adequacy of PNC. Initially, univariate logistic regression was used to calculate crude odds ratios (OR). In the multivariate analysis, all variables potentially associated with PNC adequacy were included and stratification by mode of delivery was used. A stepwise procedure was used for selecting joint variables associated with PNC adequacy, with variables being included and excluded at each step based on the likelihood ratio test. The p-values lower than 0.05 were considered statistically significant.

### Degree of Adequacy to Prenatal Care

The degree of adequacy to PNC was calculated and interpreted considering what the ordinance of the Stork Network combined with the Carvalho & Novaes Index proposed as good practice, as per the current BMoH guidelines^6,10^. It was classified into 7 categories: 1) Did not attend prenatal care: those cases in which no PNC was performed; 2) Inadequate: PNC initiated after the 12th gestational week, and pregnant women who, despite having initiated PNC until the 12th gestational week, went to less than 3 appointments; 3) Intermediate: PNC initiated up to the 12th gestational week and with attendance at 3 to 5 appointments; 4) Adequate: PNC initiated up to the 12th gestational week with attendance at 6 appointments; More than adequate: PNC initiated up to the 12th gestational week with attendance at 7 consultations or more.

## RESULTS

### General characteristics

From an estimated sample size of 14,300 childbearing women, 14,248 (99.64%) individuals aged 15 to 49 years were enrolled in the study; 816 (5.70%) were excluded due to lack of information regarding age, origin (municipality), or for not responding to the variable ‘gestational age,’ which was used to calculate the criteria of adequacy to the PNC. Thus, 13,432 (93.99%) childbearing women across RS were included in the study, of which 13,211 performed at least one prenatal consultation and 221 did not undergo PNC. Note that women in the categories of not adequate PNC (did not attend prenatal care / Inadequate / Intermediate) presented statistically significant lower levels of education when compared with women with adequate and more than adequate PNC (p = 0.000).

Table 1 shows the general characteristics of the study population according to prenatal care adequacy.

**Table 1:** Sociodemographic characteristics of the study population according to prenatal care adequacy (n = 13,432).

Sociodemographic characteristics	Did not attend or had an inadequate and intermediate prenatal care ^a^		Had an adequate and more than adequate prenatal care ^b^	
	
	(n = 5,715)		(n = 7,717)	
	
	n	%	n	%
Age group (years)				
15–19	936	16.4	810	10.5
20–24	1,673	29.3	1,933	25.1
25–29	1,303	22.8	2,008	26.0
30–34	976	17.1	1,714	22.2
35–39	629	11.0	990	12.8
≥ 40	198	3.5	261	3.4
Race/ethnic group				
White	4,187	73.3	6,225	80.7
Mixed race	701	12.3	586	7.6
Black	7	0.1	7	0.1
Indigenous	687	12.0	738	9.6
Yellow	34	0.6	34	0.4
Not reported	99	1.7	127	1.6
Level of education				
Illiterate	8	0.1	1	0.0
Partial elementary	1,378	24.1	1,118	14.5
Completed elementary	985	17.2	1,079	14.0
Partial high school	946	16.6	1,12	14.5
Completed high school	1,625	28.4	2,891	37.5
Partial higher education	278	4.9	472	6.1
Completed higher education	358	6.3	898	11.6
Not reported	135	2.4	138	1.8

^a^Did not attend prenatal care: those cases in which no prenatal care was performed. Inadequate: prenatal care initiated after the 12th gestational week, and pregnant women who, despite having initiated prenatal care until the 12th gestational week, went to less than 3 appointments. Intermediate: prenatal care initiated up to the 12th gestational week and with attendance at 3 to 5 appointments.

^b^Adequate: prenatal care initiated up to the 12th gestational week and that had an adequate number of appointments (100%) for gestational age at birth, considering the schedule of 6 appointments. More than adequate: prenatal care initiated up to the 12th gestational week with attendance to 7 appointments or more.

The PNC coverage in the study population was 98.4% (13,211/13,432), which means, the attendance of at least one PNC consultation during the pregnancy period.

The mean gestational age at the first visit was 12.5 weeks (SD: 6.49).

Of the 13,211 childbearing women who underwent PNC, 83.9% (n = 11,087) had at least 6 consultations, 75.2% (n = 9,937) had at least 7 consultations, 63.5% (n = 8,396) had ≥ 8 consultations. Approximately 16% (n = 2,124) of the women attended 5 consultations or less.

At the time of hospital admission for delivery, 99.7% (13,173/13,211) of the women brought their prenatal cards, 0.3% (36/13,211) had the card but did not bring it, and two parturients did not have the prenatal card.

The analysis of previous pregnancies showed that 39.3% (5,191/13,211) of the childbearing women were in their first pregnancy, 30.0% (3,970/13,211) were in their second pregnancy, 16.3% (2,157/13,211) were in their third pregnancy, and 14,4% (1,812/13,211) were in their fourth pregnancy or more.

Regarding the number of previous deliveries, 15,6% (2,060/13,211), had no history of previous deliveries, 40.7% (5,375/13,211) had one previous delivery, 24.6% (3,245/13,211) had 2 previous deliveries, 11.2% (1,483/13,211) had 3 previous deliveries, and 7.9% (1,043/13,211) had 4 or more previous deliveries.

Regarding the mode of delivery in the current pregnancy, 57.2% (n = 7,679) had a cesarean section, 42.8% (n = 5,744) had a vaginal delivery, and 0.04% (n = 5) had no reported data. Table 2 shows data about mode of delivery stratified by macro-regions of residence. The mean gestational age at birth was 38.5 weeks (13,277/13,432, SD: 1.9).

**Table 2:** Mode of delivery stratified by the childbearing women’s macro-region of residence.

Macro-region	Vaginal			Cesarean		
	
	n	%	95%CI	n	%	95%CI
Midwest	357	32.7	29.9–35.5	732	67.0	64.1–69.7
Metropolitan	3,317	55.1	53.9–56.4	2,701	44.9	43.6–46.1
Missionary	231	21.2	18.8–23.6	860	78.8	76.4–81.2
North	468	33.6	31.2–36.2	922	66.3	63.8–68.8
Sierra	521	35.8	33.3–38.3	934	64.2	61.7–66.7
South	525	41.0	38.3–43.6	757	59.0	56.4–61.7
Valleys	325	29.6	26.9–32.3	773	70.4	67.7–73.1

CI: confidence interval.

An analysis of the availability of laboratory tests for diagnosing HIV and syphilis showed that 96.0% (12,687/13,211) of the childbearing women had at least one anti-HIV test, 55.8% (7,373/13,208) one rapid test for syphilis, and 75.0% (9,895/13,199) one rapid test for syphilis and one VDRL during the pregnancy.

At delivery, 98.1% (13,181/13,432) were tested for HIV (using an HIV rapid test), and 96.9% (13,015/13,432) for syphilis. Among those tested for syphilis, 58.4% (7,595/13,015) were offered a rapid test, 45.9% (5,977/13,015) a VDRL, 0.1% (8/13,015) an immunofluorescence FTA-Abs test, and 0.8% (107/13,015) performed other diagnostic tests.

The analysis of the adequacy of PNC in the RS and in the state’s seven health macro-regions following the guidelines of the BMoH (Table 3) showed that, at the state level, 57.5% (n = 7,717, 95%CI: 56.3–58.6) started PNC up to the 12th of pregnancy and had at least six consultations. Also, according to BMoH criteria, the degree of inadequacy of PNC in the state reached 37.6% (n = 5,054, 95%CI: 36.8–38.5), being more expressive in the Metropolitan region (39.2%, n = 2,359, 95%CI: 37.9–40.4) and in the Sierra region (39.1%, n = 570, 95%CI: 36.6–41.6). Considering the macro-regions, the Midwest macro-region contributed to the highest percentage of non-PNC attendance, with 2.9% (n = 32, 95%CI: 1.9–3.9), followed by the Metropolitan macro-region, with 2.4% (n = 146, 95%CI: 2.0–32.8), and the South, with 1.1% (n = 14, 95%CI: 0.5–1.7).

**Table 3:** Prenatal care adequacy in the state’s seven health macro-regions, Rio Grande do Sul, Brazil.

Variables	Did not attend		Inadequate ^b^		Intermediate ^c^		Adequate ^d^		More than adequate ^e^	

	prenatal care ^a^									

	n	% (95%CI)	n	% (95%CI)	n	% (95%CI)	n	% (95%CI)	n	% (95%CI)
State										
Rio Grande do Sul	221	1.6 (1.4–1.9)	5,054	37.6 (36.8–38.5)	440	3.3 (3.0–3.6)	440	3.3 (3.0–3.6)	7,277	54.2 (53.3–55.0)
Macro-regions										
Midwest	32	2.9 (1.9–3.9)	400	36.6 (33.7–39.5)	23	2.1 (1.3–3.0)	39	3.6 (2.5–4.8)	600	54.8 (51.8–57.7)
Metropolitan	146	2.4 (2.0–2.8)	2,357	39.2 (37.9–40.4)	265	4.4 (3.9–4.9)	199	3.3 (2.9–3.8)	3,051	50.7 (49.4–52.0)
Missionary	3	0.3 (0.0–0.6)	419	38.4 (35.5–41.3)	27	2.5 (1.5–3.4)	34	3.1 (2.1–4.1)	608	55.8 (52.8–58.7)
North	13	1.0 (0.4–1.5)	508	36.5 (34.0–39.1)	44	3.1 (2.2–4.0)	58	4.2 (3.2–5.3)	769	55.2 (52.6–57.8)
Sierra	8	0.5 (0.2–0.9)	569	39.1 (36.6–41.6)	25	1.7 (1.0–2.4)	44	3.0 (2.1–3.9)	809	55.6 (53.1–58.2)
South	14	1.1 (0.5–1.7)	489	38.2 (35.5–40.8)	40	3.1 (2.1–4.0)	37	2.9 (2.0–3.8)	703	54.8 (52.0–57.5)
Valleys	5	0.5 (0.1–0.9)	312	28.3 (25.7–31.0)	16	1.5 (0.8–2.2)	26	2.4 (1.5–3.4)	740	67.3 (64.5–70.0)

CI: confidence interval.

^a^ Cases without prenatal care.

^b^ Prenatal care initiated after the 12th gestational week; and pregnant women who, despite having initiated prenatal care until the 12th gestational week, went to less than 3 appointments.

^c^ Prenatal care initiated up to the 12th gestational week and with attendance at 3 to 5 appointments.

^d^ Prenatal care initiated up to the 12th gestational week and that had an adequate number of appointments (100%) for gestational age at birth, considering the schedule of 6 appointments.

^e^ Prenatal care initiated up to the 12th gestational week with attendance at 7 appointments or more.

Table 4 shows the multivariate analysis of factors associated with adequate and more than adequate PNC by mode of delivery in the RS, Brazil. In the univariate model, all variables (age, race/ethnicity, level of education, previous pregnancies, previous deliveries, and macro-region of residence) were associated with the adequacy of PNC. All variables remained statistically significant in the multivariate model for both modes of delivery, except for the variable ‘previous pregnancies.’ For each 1-year increase in the childbearing woman’s age, the chance of an adequate PNC (OR = 1.03 [1.02–1.04]) increased 3% for women who performed cesarean section whereas, for those with vaginal delivery, it increased 5% (OR = 1.05 [1.04–1.06]). Women with cesarean delivery and no history of previous births were 145% more likely to have an adequate PNC when compared with the ones with 3 or more previous deliveries (OR = 2.45 [2.03–2.95]) whereas women with vaginal delivery were 166% more likely to have an adequate PNC. Regardless of the mode of delivery, women from the macro-region of the Valleys are 66% more likely to have an adequate PNC when compared with the ones residing in the Metropolitan macro-region (OR = 1.66 [1.43–1.92]) (data not shown).

**Table 4:** Multivariate analysis of factors associated to adequate and more than adequate prenatal care by mode of delivery, Rio Grande do Sul, Brazil.

Variables	Mode of delivery			

	Vaginal		Cesarean	
	
	OR^a^ (95%CI)	p-value	OR^a^ (95%CI)	p-value
Parturient woman age (increment of 1 year)^b^	1.05 (1.04–1.06)	0.000	1.03 (1.02–1.04)	0.000
Race/Ethnic group				
White	1.16 (1.02–1.32)	0.029	1.30 (1.15–1.47)	0.000
Non-White	1		1	
Level of education				
Illiterate and partial elementary	1		1	
Completed elementary and partial high school	1.43 (1.23–1.66)	0.000	1.09 (0.94–1.26)	0.276
Completed high school	1.72 (1.47–2.02)	0.000	1.40 (1.21–1.63)	0.000
Partial and completed higher education	1.48 (1.18–1.86)	0.001	1.47 (1.23–1.76)	0.000
Previous deliveries				
0	2.66 (2.14–3.32)	0.000	2.45 (2.03–2.95)	0.000
1	2.06 (1.73–2.45)	0.000	1.90 (1.63–2.21)	0.000
2	1.41 (1.19–1.67)	0.000	1.55 (1.33–1.81)	0.000
≥ 3	1		1	
Macro-region				
Metropolitan	1		1	
Midwest	1.43 (1.13–1.82)	0.003	0.98 (0.82–1.17)	0.784
Missionary	0.97 (0.73–1.28)	0.809	0.92 (0.78–1.09)	0.333
North	1.17 (0.96–1.44)	0.128	1.07 (0.91–1.26)	0.385
Sierra	1.20 (0.99–1.46)	0.063	1.02 (0.87–1.20)	0.787
South	1.30 (1.07–1.58)	0.008	0.98 (0.83–1.16)	0.828
Valleys	1.72 (1.34–2.21)	0.000	1.57 (1.31–1.89)	0.000

OR: odds ratio; 95%CI: 95% confidence interval.

^a^ OR adjusted by all other variables in the model.

^b^ Increase of 1 year in the age of the childbearing woman considering the initial age of 15 years old.

## DISCUSSION

The results of this study show an almost universal PNC coverage among women using SUS in the state of RS considering at least one prenatal appointment.

Data from the *Sistema de Informações sobre Nascidos Vivos* (SINASC – Live Birth Information System) show the evolution of PNC coverage in Brazil, where, the percentage of Brazilian pregnant women who have not had any prenatal consultations drops year after year, ranging from 1.92% in 2017 to 1.70% in 2018 and 1.52% in 2019^12^. The RS follows the national trend of reducing the number of pregnant women who do not have any prenatal consultations, from 1.81% in 2017, to 1.32% in 2018 and 1.15% in 2019^12^. Likewise, RS shows an expansion of prenatal coverage of women having 7 consultations or more, in 2017, it was 76.46% , increasing to 78.72% in 2018, and reaching almost 80% in 2019^12^. The percentage of lack of PNC during the study period is similar to that available on SINASC and other studies nationwide^13,14^.

Although the study data has demonstrated a very high PNC coverage in the RS, only half of the women residing in RS received adequate PNC according to the minimal criteria established by the BMoH. Other authors who considered other components of PNC service offered besides the timing of PNC onset and the number of appointments found similar results^15,16^. Leal et al.^14^, published data from the study “*Birth in Brazil,*” where the nationwide prevalence of women with early prenatal onset was 56.5%, whereas the South region (comprised of Rio Grande do Sul, Santa Catarina, and Paraná) had a prevalence of 57.6 % and the highest coverage of women with at least six prenatal appointments, with 77.3%. These authors found an overall PNC adequacy for women in the South region of Brazil of 66.7% when considering women with and without gestational complications^14^.

Women with more previous pregnancies also had less PNC appointments in the current pregnancy, whereas young women with low levels of education are those who did not attend PNC visits. Domingues et al.^15^, in a nationwide hospital-based study, observed a higher PNC inadequacy proportion in black adolescent women, with lower levels of education and multiparous in the North and Northeast regions of the country. As previously described, women who use public services have a different profile, with a higher proportion of pregnant adolescent women, of lower economic class, multiparous, and without a partner, factors associated with a later PNC onset, leading to a lower degree of adequacy in the number of consultations, exams, and orientations^16^. Also, evidence has suggested that the mother’s level of education is one of the major determinants that contribute to the late PNC onset, as well as socioeconomic status, but other factors, such as availability, accessibility, acceptability, family support, and previous experiences with the health system may also affect the timing of the first PNC appointment^19^.

Data obtained in our study regarding the maternal characteristics age, white ethnicity^14,20^, educational levels^17^, age group^14,17,20^, gestational age at first PNC visit^21^, gestational age at birth^21^, and number of previous pregnancies^14,17^ is in accordance to other studies published.

Some studies have reported better PNC adequacy in regions with better Municipal Human Development Index (MHDI), whereas others have tried to associated it with the Family Health Strategy (FHS) availability or the municipalities size and high income^13,18,22,23^. Benzaken et al.^13^ demonstrated a positive correlation between PNC adequacy with the MHDI, since the Brazilian capital cities with higher MHDI showed higher adequacy. Cunha et al.^23^, observed that municipalities with FHS and large-sized (with more than 50,000 inhabitants) presented lower PNC adequacy when compared with municipalities with FHS and up to 10,000 inhabitants. Distinctively, Tomasi et al.^18^ has shown adequate PNC in large-sized municipalities (more than 300,000 inhabitants) with not only FHS coverage of 100% but also higher MHDI levels (0.788–0.919).

The adequate PNC proportion in our study was significantly different between the State’s macro-regions. The Valleys macro-region showed the highest adequacy prevalence whereas the Metropolitan and the Sierra macro-regions presented the lowest ones. Although these macro-regions have both high MHDI and available FHS, note that Sierra and Metropolitan macro-regions have many so-called ‘dormitory towns,’ which could explain the lowest PNC adequacy prevalence, especially if we consider the barriers in the access to centers for diagnosis and treatment, the business hours that these centers remain open, the geographical difficulties, and the large distances these women may face to get to the health services.

Regarding the routine exams, the national guidelines recommend two serological tests for syphilis and HIV diagnosis. The testing service in childbearing women in the RS was efficient, notably regarding testing at the moment of delivery. A recent published analysis has reported that the coverage of at least one VDRL test and one HIV test during pregnancy, in Brazil, was 88% and 79%, respectively, with the South region showing the highest rate of testing performance^14^. In the South region, the coverage of at least one VDRL test during pregnancy was 94.7% whereas for HIV it was 92.2%^14^.

The proportion of women in possession of their prenatal card was high. Similar results were described in studies carried out in the city of Pelotas, which belongs to the South macro-region of the RS, whose prevalence of possession of the prenatal card was 83%^24^, whereas it was 72% in a Brazilian national hospital-based survey^15^. Some reports also differ from our findings. Gonzalez et al^25^., in a study involving 10,242 pregnant women in the city of Rio Grande, RS, which also belongs to the South macro-region, found that only 54.8% of the childbearing women had the prenatal card, but also, with notably higher possession among puerperal women assisted in the public service compared with those in the private sector (62% *versus* 44%)^25^.

Another outstanding data were the cesarean rates performed in the RS. The ideal cesarean rate, adjusted for the Brazilian population, according to the WHO, ideally ranges from 25%–30%^26^, but in our study, in a state level, the cesarean rate found was twice higher than the one recommended, with the highest rates being observed in the macro-regions of Missionary and Valleys, almost three times higher than the ideal.

Cesarean rates have been increasing even for low obstetric risk births, especially among women with higher educational levels^27^. The Metropolitan (55.1%) and South (41.0%) macro-regions showed the highest rates of vaginal delivery, but rates are still low when considering the BMoH and the WHO recommendations. Studies have shown that the option for cesarean delivery is more frequent in private institutions (80–90%) than in public ones (35–45%)^28,29^, and an almost universal use of c-sections has been reported for births of wealthier, white women, with higher educational level and with greater adequacy to PNC in private health facilities in Brazil^27,30^. Differently than previously reported, what our study observed is a preference for cesarean delivery in SUS maternity hospitals in the state of RS for a reason yet to be described, and it is widespread, since high c-section rates were observed in all macro-regions of the state.

Also note that the data from this manuscript is from a covid-19 pre-pandemic period. The SARS-CoV-2 pandemic may have greatly impacted prenatal management and surveillance and raised the need for clear tailored guidelines for addressing significant health disparities in maternal care access and pregnancy outcomes in the RS, thus further research considering the effects of the pandemic in this population is also advised.

Also, this study had some limitations: a) it is a cross-sectional study based on secondary data collection that depends on the completeness and availability of information at the time of collection; b) there is a possibility of an ecological fallacy when analyzing aggregate data; c) it lacks information regarding pregnancy outcomes; d) it cannot assess the adequacy of each PNC visit content; and e) it considered low-risk and high-risk pregnancies indistinctly, but high-risk pregnancies require more visits, even with late initiation.

Nevertheless, these limitations were minimized by the use of standardized data collection instruments, the large size of the sample, and the study design applied. Further research that considers the pregnancy outcomes is advised. Also, analyzing the adequacy considering high-risk and low-risk pregnancy is recommended.

In fact, the number of consultations and the onset timing to ensure adequate PNC are the most frequent indicators used in studies that assess PNC and it is the BMoH current recommendation. However, the assessment of adequacy based on the coverage of consultations reflects only the contact with the assistance service. In our study, despite the almost universal PNC coverage in RS, the PNC offered by the SUS was adequate only for half of the population assisted. The disparity between high coverage and inadequate use of prenatal care may be related to socioeconomic, demographic, and behavioral risk factors therefore public health policies targeted at women receiving care in this setting shall be revisited.
